# Correlation of histopathologic and dynamic tissue perfusion measurement findings in transplanted kidneys

**DOI:** 10.1186/1471-2369-14-143

**Published:** 2013-07-11

**Authors:** Thomas Scholbach, Hsin-Kai Wang, An-Hang Yang, Che-Chuan Loong, Tsai-Hong Wu

**Affiliations:** 1 Hospital for Children and Adolescents, Chemnitz Clinics, Flemmingstr. 4, D −09116, Chemnitz, Germany; 2 Department of Radiology, Taipei Veterans General Hospital and School of Medicine, National Yang-Ming University, Taipei, Taiwan R.O.C; 3 Department of Pathology, Taipei Veterans General Hospital and School of Medicine, National Yang-Ming University, Taipei, Taiwan R.O.C; 4 Department of Surgery, Taipei Veterans General Hospital and School of Medicine, National Yang-Ming University, Taipei, Taiwan R.O.C; 5 Department of Internal Medicine, Taipei Veterans General Hospital and School of Medicine, National Yang-Ming University, Taipei, Taiwan R.O.C

## Abstract

**Background:**

Cortical perfusion of the renal transplant can be non-invasively assessed by color Doppler ultrasonography. We performed the Dynamic Tissue Perfusion Measurement (DTPM) of the transplant’s renal cortex using color Doppler ultrasonography (PixelFlux technique), and compared the results with the histopathological findings of transplant biopsies.

**Methods:**

Ninety-six DTPM studies of the renal transplant’s cortex followed by transplant biopsies were performed in 78 patients. The cortical perfusion data were compared with the parameter of peritubular inflammatory cell accumulation (PTC 0 to 3) based on Banff-classification system.

**Results:**

A significant decrease of cortical perfusion could be demonstrated as the inflammatory cells accumulation in peritubular capillaries increased. Increasing peritubulitis caused a perfusion loss from central to distal layers of 79% in PTC 0, of 85% in PTC 1, of 94% in PTC 2, and of 94% in PTC 3. Furthermore, the perfusion loss due to peritubular inflammation was more prominent in the distal cortical layer. The extent of perfusion decline with increasing peritubulitis (from PTC 0 to PTC 3) was 64% in proximal 20% cortical layer (p20), 63% in proximal 50% cortical layer (p50), increased to 76% in distal 50% cortical layer (d50), and peaked at 90% in the distal 20% cortical layer (d20). For those without peritubulitis (PTC 0), the increase in the the Interstitial Fibrosis/Tubular Atrophy (IF/TA) score was accompanied by a significantly increased cortical perfusion. A Polyomavirus infection was associated with an increased cortical perfusion.

**Conclusions:**

Our study demonstrated that the perfusion of the renal transplant is associated with certain pathological changes within the graft. DTPM showed a significant reduction of cortical perfusion in the transplant renal cortex related to peritubular capillary inflammation.

## Background

Acute and chronic damage to renal transplants requires an early and accurate diagnosis to start the appropriate treatment. Renal transplant biopsies are an established method to evaluate such changes. These biopsies are performed as routine protocol biopsies in regular intervals in asymptomatic recipients or are performed when a clinical demand emerges. Biopsies are invasive and therefore a need for less potentially harmful procedures exists. Sonography is widely used to describe morphological changes of the transplant. Doppler sonography is used to describe changes of the blood flow to transplants. Today, only limited information can be drawn from Doppler sonographic measurements. To refine the impression of a transplant’s perfusion contrast enhanced sonography was introduced. Nevertheless, a need for a more differentiated evaluation remains. Dynamic tissue perfusion measurement (DTPM) has demonstrated the potential to describe perfusion in renal cortex in thin cortical slices with more than 50 parameters [1-5] as well as in other organs [6-10].

### Aim

Our aim was to compare histopathological descriptions of renal transplants with DTPM data to plumb the potential of this new tool.

## Material

This study was approved by Institutional Review Board. Totally, 96 DTPM studies and renal transplant biopsies were performed in 78 Taiwanese patients (single study for 63 patients; two studies for 12 patients; three studies for 3 patients) between December 2005 and September 2008. The mean duration between the patient receiving renal transplantation and DTPM study was 44.8 months. Among 78 patients, there were 41 male and 37 female; the mean age when receiving living or deceased donors’ renal transplantation was 41.0 ± 12.6 years (range 6–72 years); 68 received renal transplantation from deceased donor, 6 received simultaneous pancreas and kidney transplantation, 4 received living-related renal transplantation. The indication for grafting was end stage renal disease; the transplant surgery was performed in Taiwan or People’s Republic of China. Details of the transplant recipients are outlined below:

## Methods

### Patient consent

Informed consent was obtained from every patient before the study.

### Ethical approval

This study was supported by research grants 96-2321-B-075-009 and 97-2314-B-075-052 from the National Science Council, Taiwan and was approved by the Institutional Review Board, Taipei Veterans Genereal Hospital, Taipei, Taiwan.

### Color doppler sonography and dynamic tissue perfusion measurement

**Figure 1:**
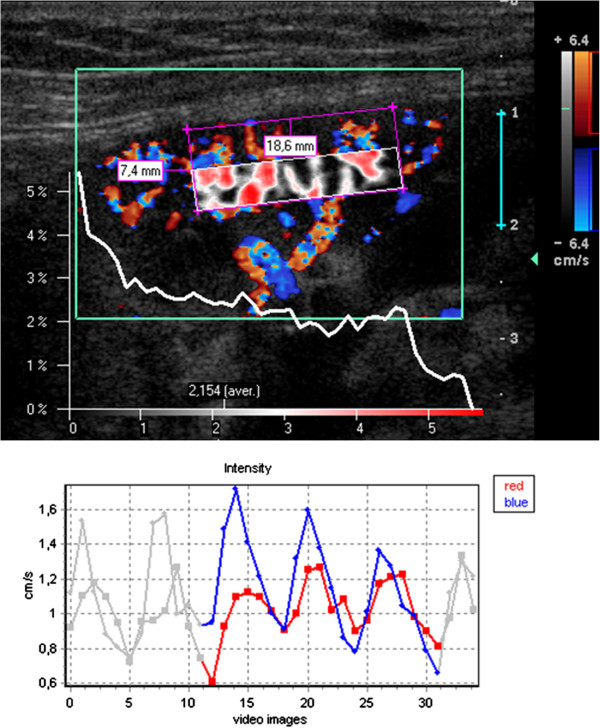
**False color map of tissue perfusion of the proximal 50% of the renal cortex (sub**-**ROI p50) and perfusion intensity distribution diagram.** Below: time curve of perfusion intensity with 3 heart cycles highlighted for calculation. Colors of the lines correspond to the respective colors inside the color Doppler sonographic video: blue (red) line – course of the intensity of blue (red) pixels).

**Figure 2:**
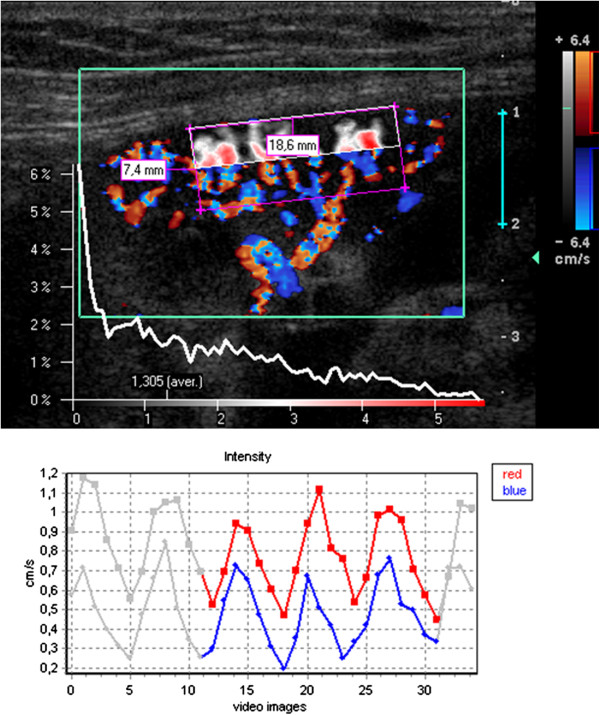
**False color map of tissue perfusion of the distal 50% of the renal cortex (sub**-**ROI d50) and perfusion intensity distribution diagram.** Below: time curve of perfusion intensity with 3 heart cycles highlighted for calculation.

Each transplanted kidney was scanned by means of color Doppler Sonography by a single investigator (H.-K. W.) within one day prior to transplant biopsy. An ATL HDI-5000 ultrasound unit (Philips Medical System, Bothell, WA, USA) equipped with a linear L12-5 MHz transducer was used for color Doppler study. A standardized preset of the imaging parameter was used during the whole course of imaging acquisition: imaging depth, 4 cm; color Doppler frequency, 6 MHz; pulse repetition frequency, 1000 Hz; color gain, 79%; wall filter, medium; flow option, medium velocity; color map, 3. A longitudinal section of the transplanted kidney encompassing the outer cortex was selected for scanning. The imaging field remained unchanged during scanning. The pressure of the ultrasound transducer to the skin was kept minimal. Two color Doppler cine loops video encompassing at least two full heart cycles (40 frames with a frame rate of 9–10 images/sec) were recorded and stored in Digital Imaging and Communications in Medicine (DICOM) format. All dynamic tissue perfusion measurements of the recorded color Doppler cine videos using the software PixelFlux (Chameleon-Software 2009) were performed by a single investigator (T.S.) blinded to the histopathological results. The perfusion measurement of the entire region of interest (ROI) in one cine loop took about 10 seconds. The central segment of the outer cortex was selected for dynamic tissue perfusion measurement (its steps are listed in Table 1). In each patient 14 single measurements of each video clip were made. Ten of these measurements encompassed 10% of the ROI in a sequence from the proximal to the distal cortical layers and 4 measurements encompassed wider sections (20 and 50% of the proximal and distal cortex (Figures 1 and 2 demonstrating an example of perfusion measurement in the proximal and distal 50% of the cortex). Altogether the entire ROI was completely measured two times in each video with at different pattern of slicing. Figures 1 and 2 demonstrate the setting of the ROI and the selection of a sub-ROI in slices. The corners of the parallelogram lie on the watershed between two neighboring vascular segments. They define the lateral borders of the ROI. Two corners on the outer edge of the medullary pyramids define the proximal border whereas the other two corners are set on the renal surface. This way the distal border of the ROI is defined by the line between these two corners. Figures 1 and 2 demonstrate the perfusion of the proximal and distal cortex with a false color map of the perfusion distribution, an intensity distribution diagram, below (showing clear differences between proximal and distal ROI) and the change of the perfusion intensity during the heart cycle, respectively.

**Table 1:** **Workflow chart for Dynamic Tissue Perfusion Measurement** (**DTPM**) **of transplant kidneys**

Step 1	The software opened the video and calibrated the distances automatically	
Step 2	The software read out the color bar and all hues were calibrated	
Step 3	Maximal velocities encoded by the colors were registered	
Step 4	Selection of the region of interest (ROI)	a. The ROI consisted of a parallelogram which included an entire cortical segment in the center of the transplant, fed by one interlobar artery running straight towards the transducer.
		b. The four corners of the parallelogram were set as follows:
		i. At the center of the outer edge of a medullary pyramid (1)
		ii. At the center of the outer edge of the neighboring medullary pyramid (2)
		iii. At the renal surface perpendicular to the first two corners (3 and 4)
		c. The ROI was sliced into defined horizontal slices
		i. Each slice stretched from the left to the right border of the ROI and had a height which encompassed a certain percentage of the ROI’s entire height (10%, 20% , 50% or 100%). So the sub-ROI encompassing the proximal 20% (resp. 50%) was labeled p20 (resp. p50) and those encompassing the distal 20% (resp. 50%) was labeled d20 (resp. d50). (Figures 1 and 2)
		ii. These slices were arranged to cover adjacent horizontal strips of the ROI.
Step 5	Dynamic tissue perfusion measurement was initiated. All steps (a-f) were carried out automatically by the PixelFlux-software	a. Each pixel (colored or grey) was evaluated with respect to its size and coloration.
		b. The color bar’s hues were assigned a velocity value according to its relative distance from zero to the maximum velocity value.
		c. The relative distance value (from 0 to 1) was then multiplied by the maximum velocity indicated to calculate a specific velocity value for each color hue. Colorless pixels were assigned the velocity of zero.
		d. The mean velocity value of all colored pixels inside the ROI and the area of all colored pixels were calculated.
		e. The measurements were repeated for all consecutive images of the video.
		f. The software recognized beginning and end of any heart cycle inside the video automatically and restricted all consecutive calculations to a full heart cycle.
		g. Then the mean perfusion velocity of each image was multiplied by the mean perfused area. This product was divided by the area of the entire ROI resulting in the mean flow intensity of the ROI during a complete heart cycle.

### Biopsies

Transplant biopsies were performed under sonographic-guidance by a single investigator (H.-K. W). An 18-gauge core biopsy needle (Temno, Cardinal Health, McGaw Park, IL, USA) was used to sample tissue from the outer cortex including parts of the cortico-medullary junction of the transplanted kidney. The specimens were formalin-fixed for light microscopy; glutaraldehyde-fixed for electron microscopy; or placed in phosphate buffered solution for immunofluorescent microscopy.

### Histology

All biopsy specimens were evaluated by a pathologist specialized in renal transplant pathology (A.-H, Y.). Classification of peritubular inflammation (PTCs), interstitial fibrosis (IF/TA), and polyomavirus infection (BKN) were based on the Banff scoring criteria revised in 2003 [11,12], 15, 16) (Table 2).

**Table 2:** **Banff classification of inflammatory cells in peritubular capillaries** (**PTCs**), **degree of interstitial fibrosis** (**IF**/**TA**), **and stages of polyomavirus infection** (**BKN**)

PTC 0	No significant cortical peritubular inflammatory changes
PTC 1	Cortical peritubular capillary with 3–4 luminal inflammatory cells
PTC 2	Cortical peritubular capillary with 5–10 luminal inflammatory cells
PTC 3	Cortical peritubular capillary with >10 luminal inflammatory cells
IF/TA 0	No interstitial fibrosis and tubular atrophy
IF/TA 1	Mild interstitial fibrosis and tubular atrophy (<25% of cortical area)
IF/TA 2	Moderate interstitial fibrosis and tubular atrophy (26–50% of cortical area)
IF/TA 3	Severe interstitial fibrosis and tubular atrophy/ loss (>50% of cortical area)
BKN- stage A	Viral activation in cortex and/or medulla with intranuclear inclusion bodies AND/OR positive immunohistochemistry or in situ hybridization signals, no or minimal tubular epithelial cell necrosis/lysis, no denudation of tubular basement membranes, no or minimal interstitial inflammation in foci with viral activation, no or minimal tubular atrophy and interstitial fibrosis
BKN- stage B	Marked viral activation in cortex and/or medulla, marked virally induced tubular epithelial cell necrosis/lysis and associated denudation of tubular basement membranes, interstitial inflammation, interstitial fibrosis and tubular atrophy

### Statistics

Data were explored statistically using SPSS 17 and PASW 18 software. Groups were compared applying the Mann–Whitney-U-test. P-values less than 0.05 were regarded statistically significant.

## Results

Among 96 DTPM studies, 36 belonged to the PTC 0-group, 46 to the PTC 1-group, 11 to the PTC 2-group and 3 to the PTC 3-group; 47 belonged to the IF/TA 0-group, 32 to the IF/TA 1-group, 17 to the IF/TA 2-group and 0 to the IF/TA 3-group.

**Figure 3:**
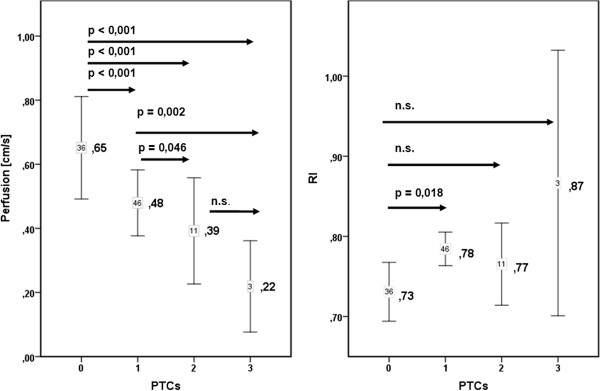
**Significant decline of cortical perfusion in DTPM-measurements (left) compared to insignificant increase of traditional RI measurements (right) in transplants with increasing peritubular inflammation.** Significant differences are indicated by p-values (n.s. - not significant). Score for increasing peritubular inflammation: PTC 0 to PTC 3.

The mean perfusion intensities were 0.65 (cm/s) in PTC 0, 0.48 in PTC 1 (p < 0.001 vs. PTC 0), 0.39 in PTC 2 (p < 0.001 vs. PTC 0, p = 0.046 vs. PTC1), 0.22 in PTC 3 (p < 0.001 vs. PTC 0, p = 0.002 vs. PTC 1) (Figure 3, Tables 3 and 4). The conventional RI (resistance index) were 0.73 in PTC 0, 0.78 in PTC 1 (p = 0.018 vs. PTC 0), 0.77 in PTC 2, 0.87 in PTC 3 (Figure 3, Table 3). RI showed a weaker connection to peritubular inflammation than did cortical tissue perfusion with only one significant difference between the PTC-classes compared to five significant differences among the DTPM measurements (Figure 3).

**Table 3:** **Mean values**, **numbers** (**N**) **and standard deviations** (**SD**) **of global cortical perfusion intensity in DTPM and resistance indices** (**RI**) **in groups with different PTC scores**

**Perfusion intensity [cm/s]**				**RI**		
**PTCs**	**Mean**	**N**	**SD**	**Mean**	**N**	**SD**
0	0,65	504	0,617	0.73	36	0.112
1	0,48	644	0,468	0.78	46	0.073
2	0,39	154	0,405	0.77	11	0.087
3	0,22	42	0,194	0.87	3	0.146

**Table 4:** **p**-**values of Mann**–**Whitney**-**U**-**tests for differences of global cortical perfusion intensity in DTPM between different PTC scores**

**Perfusion intensity [cm/s]: differences between PTC groups**			
**p-values**	**PTC 0**	**PTC 1**	**PTC 2**
PTC 1	< 0,001		
PTC 2	< 0,001	0,046	
PTC 3	< 0,001	0,002	0,081

**Figure 4:**
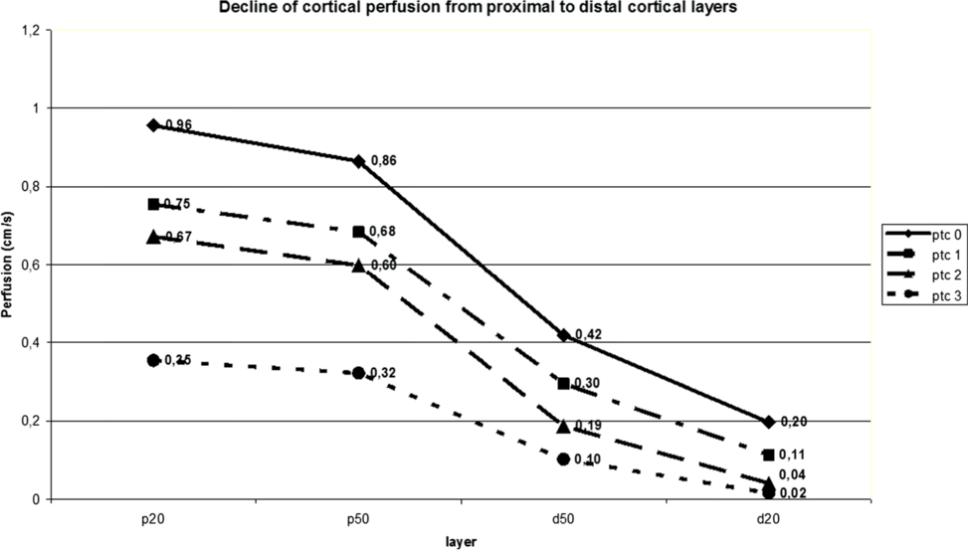
**Decline of cortical perfusion (y-axis) from proximal to distal cortical layers (x-axis) is different in the various Banff classes for peritubular inflammation (no significant cortical peritubular inflammatory changes (PTC 0) to cortical peritubular capillary with >10 luminal inflammatory cells (PTC 3)).** Cortical layers are p20: proximal 20% of the renal cortex peripheral to the medullary pyramids, p50: proximal 50%, d50: distal 50% and d20: outer 20% of the cortex.

Increasing peritubulitis caused a perfusion loss from central (p20) to distal (d20) layers from 0.96 to 0.20 (79% reduction) in PTC 0 transplants, from 0.75 to 0.11 (85% reduction) in PTC 1 transplants, from 0.67 to 0.04 (94% reduction) in PTC 2 transplants, from 0.25 to 0.02 (94% reduction) in PTC 3 transplants (Figure 4). As peritubulitis increased from PTC 0 to PTC 3, the perfusion intensity decreased from 0.96 to 0.25 (64% reduction) in p20, 0.86 to 0.32 (63% reduction) in p50, 0.42 to 0.10 (76% reduction) in d50, 0.20 to 0.02 (90% reduction) in d20 layer (Figure 4).

**Figure 5:**
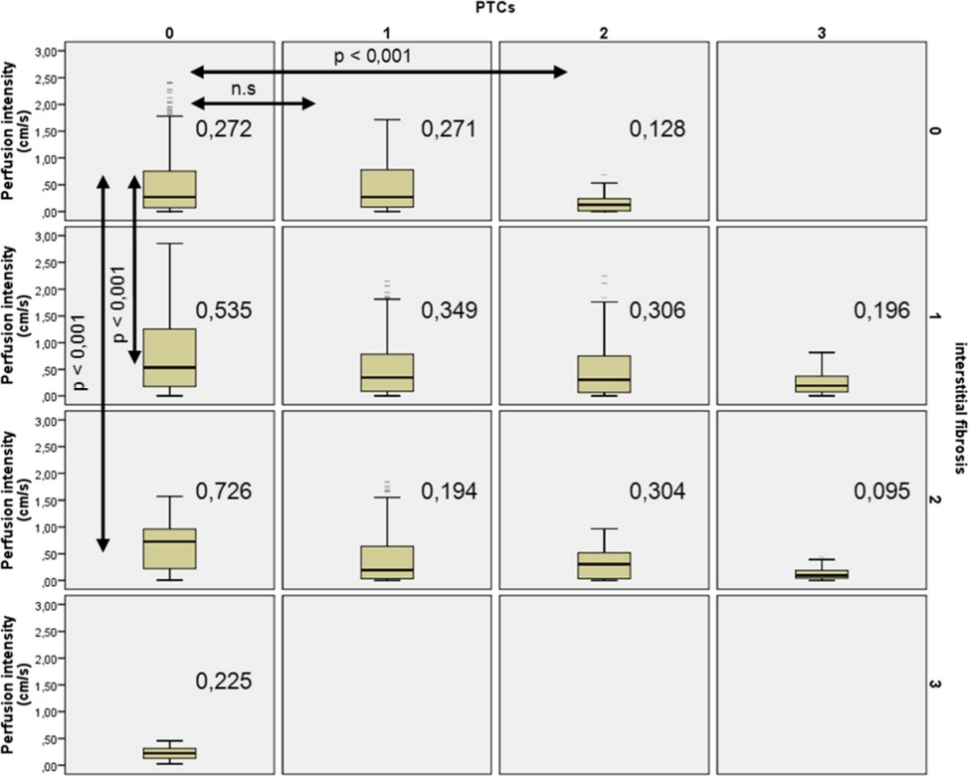
**Separate influences of peritubular inflammation (PTCs: columns) and interstitial fibrosis/tubular atrophy (rows) on the perfusion intensity of renal transplant cortices in patients with variable combinations of diverse levels of PTCs and IF/TA.** Arrows highlight comparable constellations with zero-levels of one of both parameters (boxes 01 and 10 and 02 and 20) and with constellations with one parameter at level 1 and the other at level 2 (boxes 12 and 21). P-values of Mann–Whitney –U-tests are given above the arrows. Median values for each constellation is given aside of the box plot in each box.

**Figure 6:**
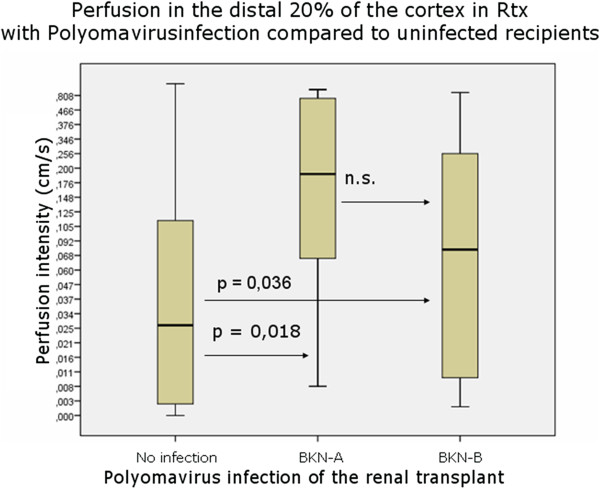
**Significant increase of cortical perfusion in Polyomavirus infection of renal transplants.** (BKN-A/B - BKN- stage A/B: see Table 2).

In those without peritubulitis (PTC 0), perfusion intensity was 0.272 in IF/TA 0, 0.535 in IF/TA 1 (p < 0.001 vs. IF/TA 0), 0.726 in IF/TA 2 (p < 0.001 vs. IF/TA 0) (Figure 5). IF/TA dependent changes were significantly higher than PTCs influences if PTCs reached values of 1 or 2 and IF/TA remained at zero-level compared to the mirror situation with IF/TA reaching levels of 1 and 2 and no PTCs (PTCs 0). Polyomavirus infection was accompanied by a significant increase in cortical perfusion in the outer cortical layer (distal 20%), albeit less pronounced in stage B than stage A (Figure 6).

## Discussion

Renal transplant biopsies are evaluated according to the Banff scoring system since it was shown that a high Banff score is predictive for the graft survival [13].

Color Doppler ultrasound of the outer renal cortex provides the basis for a quantitative measurement of the perfusion of renal transplants [1,2,14]. Vascular changes, which are mirrored by perfusion changes, may precede manifest function loss of the kidney and can be described histologically.

Peritubular inflammation is a criterion of humoral rejection in renal transplants. According to the Banff classification, this feature is scored as peritubular capillaritis (PTCs) [15]. It might be important to detect peritubular capillaritis in renal transplants early since it can precede the development of a full blown chronic antibody-mediated rejection (CAMR). Others propose to assume a chronic antibody-mediated rejection (CAMR) even in the absence of C4d deposition in cases with peritubular capillaritis, transplant glomerulitis, thickening of the peritubular capillaries basement membrane, and circulating anti-HLA antibodies [16,17]. Detection of peritubular inflammation might be significant for indicating the subsequent poor prognosis [18,19] and thus could be regarded an early sign of graft deterioration. Our study showed a significant decrease of cortical perfusion as the inflammatory cells accumulation in peritubular capillaries increased. The reduction of tissue perfusion due to peritubular inflammation depended on the position of the cortical layer too. The more distal the layer the stronger was the effect of peritubular inflammation. Cortical perfusion was also influenced by interstitial fibrosis and tubular atrophy (IF/TA). Unexpectedly, increasing IF/TA scores were connected to an increased perfusion. This might be explained by the short-circuiting of blood via shunts connecting the afferent and efferent vascular pools in juxtamedullary glomeruli [20,21]. Recently, in a mouse model the overexpression of EphB4 receptor tyrosine kinase in the kidney was shown to promote the development of such shunts [22].

Conventional color Doppler sonography with calculation of Resistance Index (RI) and Pulsatility Index (PI) were used for many years to evaluate the perfusion of renal transplants. RI measurements reflect a complex interaction of renal as well as systemic vascular influences and are therefore disregarded to be suitable for evaluation of the renal vascular state by some investigators [23,24]. An elevated RI alone cannot reliably differentiate the causes of transplanted kidney dysfunction and it is considered a nonspecific marker [25].

RI does not inform about the real blood flow velocities, since a ratio of two velocities (systolic and diastolic) is calculated. It represents spatially restricted information from a single vessel and gives no data on the width of the vessel, let alone the perfused area in a certain tissue. Thus RI is basically less suitable for describing the perfusion status of the renal parenchyma. Furthermore, the pathological changes inside the kidney can be distributed inhomogeneously [26], and RI at a single point of an artery may not represent the status of perfusion in the region of interest. In contrast, Dynamic Tissue Perfusion Measurement (DTPM) is based upon a heart cycle triggered measurement of a tissue’s mean flow velocity of all vessels inside the region of interest multiplied by the mean perfused area (respecting changing width of all vessels during the heart cycle) [3,4]. Thus, DTPM offers a more sophisticated investigation of renal perfusion by using velocity as well as perfused area information, referring all measurements to all vessels inside the tissue and is capable in differentiating all perfusion data between millimeter-thin slices. It reflects histological changes as PTCs better than conventional RI (Figure 3: left diagram compared to the right one). DTPM thus has the potential to go beyond the limitations of usual vascular resistance calculations by RI measurements. The detection of subtle perfusion changes simultaneously to a polyomavirus infection (Figure 6) clearly demonstrates that the slice wise perfusion measurement in consecutive layers of renal microvasculature offers a sophisticated access to relevant histological changes even beyond the Banff-classification. It was demonstrated in earlier investigations that with time elapsed after transplantation the cortical perfusion in renal transplants of children decreased significantly [2] and that simultaneously the pulsatility of renal transplants’ microvascular perfusion increased significantly [14]. Reduction of smaller subcapsular transplant vessels occurs in acute as well as chronic rejected organs [27]. Cortical vessel loss, as determined visually in power Doppler images, was predictive in contrary to RI as for the transplant function 1 year after transplantation [28]. Recent investigations also showed that microvascular damage is the key process linked to the loss of transplants’ function [29,30]. Our findings might be especially noteworthy since we demonstrated a significant and gradual decline of cortical perfusion along with increasing peritubular capillaritis using DTPM. DTPM clearly showed the potential to measure the progressive obliteration of the transplant’s microvessels. This process starts in the periphery of renal cortex where the vessel diameters are naturally small. Our study did not investigate the relationship of DTPM with the clinical outcome of the graft. Further studies are needed to elucidate if specific histological findings or the destruction of the microvasculature are more predictive for the transplant’s function.

In the long run many renal disorders lead to a gradual decline of cortical perfusion [31-33]. DTPM might thus be useful in other chronic diseases of the kidney too.

One limitation of our study is that we only assess the relationship between cortical perfusion and the histopathological changes of transplant kidney. Future studies are needed to confirm the role of DTPM as a surrogate of outcome of renal transplant. Then DTPM could become a non-invasive tool to reduce the need for invasive procedures such as biopsies.

## Conclusion

Microvascular perfusion changes in renal transplants can be demonstrated by DTPM. A significant reduction of cortical perfusion in the transplant kidney, especially in its peripheral layers, is related to peritubular capillary inflammation.

## Competing interests

All authors declare no competing interest.

## Authors’ contributions

TS carried out DTPM analysis, statistical analysis and drafted the manuscript. HKW carried out DTPM measurement and drafted the manuscipt. AHY carried out pathological analysis of renal transplant biopsy. CCL participated in study design and coordination. THW participated in study design and coordination. All authors read and approved the final manuscript.

## Pre-publication history

The pre-publication history for this paper can be accessed here:

http://www.biomedcentral.com/1471-2369/14/143/prepub
